# Physics-Based Artificial Neural Network Assisting in Extracting Transient Properties of Extrinsically Triggering Photoconductive Semiconductor Switches

**DOI:** 10.3390/mi15081003

**Published:** 2024-08-01

**Authors:** Zhong Zheng, Huiyong Hu, Yutian Wang, Tianlong Zhao, Qian Sun, Hui Guo

**Affiliations:** Division of Integrated Circuits, Xidian University, Xi’an 710071, China; 21111110537@stu.xidian.edu.cn (Z.Z.); huhy@xidian.edu.cn (H.H.); zhaotl@xidian.edu.cn (T.Z.); qsun_2020@stu.xidian.edu.cn (Q.S.)

**Keywords:** physics-based neural network (NN), extrinsically triggering photoconductive semiconductor switch (ET-PCSS), mixed-mode simulation, regression fitting

## Abstract

In this paper, a physics-based ANN assisting method for extracting transient properties of extrinsically triggering photoconductive semiconductor switches (ET-PCSSs) is proposed. It exploits the nonlinear mapping of ANN between transient current (input) and doping concentration (output). According to the basic laws of photoelectric device operating, two types of ANN models are constructed by gaussian and polynomial fitting. The mean absolute error (MAE) of forecasting transient photocurrent can be less than 10 A under low triggering optical powers, which verifies the feasibility of ANN assisting TCAD applied to PCSSs. The results are comparable to computation by Mixed-Mode simulation, yet even thousands of seconds of CPU runtime cost are saved in every period. To improve the robustness of the Poly-ANN predictor, Bayesian optimization (BO) is implemented for minimizing the curl deviation of photocurrent-time curves.

## 1. Introduction

Photoconductive semiconductor switches (PCSSs) are a variety of high-power pulsed switches applied in harsh fields with high voltage, large current density and ultra-high frequency such as Dielectric Wall Accelerators, missile-borne high-power microwaves and so on [1]. According to the working mode, PCSSs can be classified into two categories. One is intrinsic excitation, where electrons transition directly from a valence band to a conduction band. This is common in semi-insulating materials whose band gap is near the photon energy. The other is called extrinsically triggering (ET) by illumination with longer wavelength [2,3]. Such often happens in the form of electron transition from an impurity or from trap levels to conduction bands, for example, a recombination center, electron trap and hole trap formed by impurities. The second mode is more valuable in industrial application for its advantage of having lower requirements for light sources.

At present, ET-PCSSs based on GaAs, SiC and diamond are attracting ever-increasing interests from researchers [4,5,6,7,8,9], benefiting from their excellent photoconduction properties. In 2022, Woo et al. [5] investigated different diamond structures for extrinsic PCSSs, including type Ib (highly nitrogen-doped), IIa (unintentionally doped), boron-doped epilayer on type IIa substrate and boron-implanted type Ib and IIa substrates. In 2020, Kramer et al. [8] examined optical transitions of vanadium-doped SiC as a function of dopant concentration measured by secondary ion mass spectroscopy (SIMS). Rakheja et al. [6] built the physical modeling of intrinsic 4h-SiC PCSSs using coupled electrical and optical simulations for high-power and high-frequency applications. They adopted the numerical simulations of carrier dynamics by the Monte Carlo method. However, the extrinsic transition differs from intrinsic excitation occurring band-to-band from the perspective of photogeneration rate. Take 4H-SiC for example; the energy level and state density of impurity or traps have implicit influences on the conductive performance. In other words, what is merely known is that the vanadium acceptor levels trap the electrons donated by the nitrogen levels, which means the higher the nitrogen density is, the larger the photocurrent that can be achieved [10]. It is difficult to know the specific connection between device performance and doping profile. Unquestionably, it costs are high to explore how impurity density affects the conductive performance of PCSSs in extrinsic optical absorption (EOA) mode due to the demand of repetitive and expensive measuring, including SIMS for doping concentration and deep level transient spectroscopy (DLTS) for deep traps, especially in unintentionally doped substrates like diamond IIa and vanadium-doped semi-insulated SiC. Furthermore, PCSSs are supposed to bear tens of thousands of volts and hundreds of amperes of current within the time range of nanoseconds, or even picoseconds. It is tough to get measurements on the grounds of device reliability and environment security, especially when the bias voltage approaches the critical breakdown threshold of the 4H-SiC substrate. Hence, there are still challenges in the research on the transient properties of ET-PCSSs.

Technology Computer Aided Design (TCAD) simulation is an efficient method for extracting electrical properties [11,12,13,14,15,16], even though the devices are used in high-risk environments. In the 1990s, Joshi et al. [11] built a time-dependent drift-diffusion model to simulate the filamentary conduction of GaAs-PCSS. Kelkar [9] and Chowdhury [7], respectively, studied 2D device simulation of SiC-PCSS with and without illumination. With the development of TCAD technology, the integration of devices and circuits is gradually applied [17], such as in the Mixed-Mode simulator. Wei [14] reported the switching transience of GaAs-PCSSs under the transition from linear mode to nonlinear mode. However, according to existing experience, each run of the CPUs may take several thousands of minutes. It is harmful to efficiently optimize for parameters in different devices or simulation conditions. To this end, a state-of-the-art technology, the Artificial Neural Network (ANN), is introduced to learn the implicit nonlinear relation between photocurrent (labels) and doping concentration (features) [18]. This is the first trial on AI technology for properties forecasting of PCSSs, inspired by that for semiconductor power devices like Schottky barrier diodes (SBDs) [19,20] and Nanosheet field-effect transistors (NSFETs), as in [21,22] et al.

A trained model will have the ability to predict transient performance of photoconduction in specific PCSS devices with little time cost. That means an ANN predictor can be a substitute for TCAD simulation if the accuracy satisfies the requirement in applications. In order to make the predictor more robust, appropriate physical knowledge of the operating device becomes a priority before training an ANN model. In Section 2, a simplified physical model was established based on gauss-fitting and polynomial-fitting; thus, two types of branch models are defined. We provide the transient photocurrents under different illuminations, both by Mixed-Mode simulating and ANN forecasting. Then, experimental fittings of peak photocurrents under various optical powers are used to analyze those from ANN predictors in Section 4. Meanwhile, Bayesian optimization is applied for better structure parameters in Poly-ANN.

**Figure 1:**
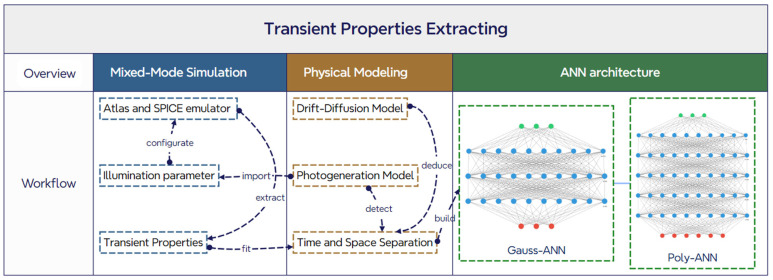
Mapping diagram of three sections: Mixed-Mode Simulation, Physical Modeling and ANN architecture.

Figure 1 demonstrates the overall flow of methods applied and relevance among three sections in this paper. In Mixed-Mode simulation, the Atlas emulator solves the physical equations for the devices, and SPICE is modeled for circuit solution. The illumination parameter imported by the photogeneration model is configurated in Atlas. In physical modeling, transient properties computed by Atlas and the SPICE mixed emulator are fitted with specific functions for time-space separation. Based on this, two types of ANN architectures are built to improve the efficiency of extracting properties of ET-PCSSs.

## 2. Physical Modeling

For semiconductor devices, the mathematical ANN predictor is limited by its lack of physical laws. Therefore, physics-based modeling can contribute to the improvement of its accuracy if proper inputs and outputs are defined in ANN models. The following will provide a simplified deduction for two kinds of physics-based models.

When PCSS devices work, the process can be considered as a directional migration of a photocarrier in an electric field. Electron–hole pairs separate following the principle of the Drift–Diffusion law shown in Equations (1) and (2) as below [23,24]:

(1) $$\vec{J_{n}}=qn\mu_{n}\nabla \vec{E}+qD_{n}\nabla n$$

(2) $$\vec{J_{p}}=qp\mu_{p}\nabla \vec{E}+qD_{p}\nabla p$$

**Figure 2:**
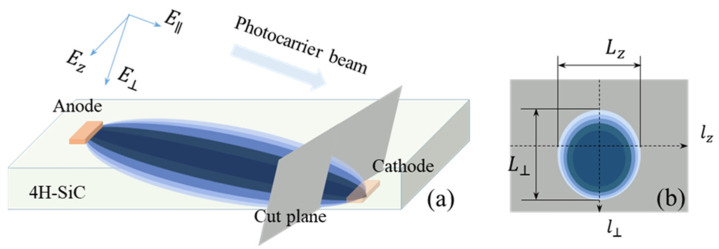
(**a**) Schematic of a photo-carrier propagating through an ET-PCSS with opposed electrodes, of which the gap spacing is 4mm and the composition is Ni/Ti/Au; (**b**) cut-plane near cathode.

Here, $\vec{J_{n}}$ is defined as current in unit area per unit time, expressed as $\vec{J_{n}}=\vec{J_{n}}\left(\sigma ,t\right)$. Similarly, $\nabla \vec{E}$, ∇n, ∇p is, respectively, the gradient of $\vec{E}\left(\sigma ,t\right)$, $n\left(\sigma ,t\right)$, $p\left(\sigma ,t\right)$. $\mu_{n}$,$D_{n}$ is the mobility and diffusion coefficient of electrons, while $\mu_{p}$, $D_{p}$ is the same for holes. As seen in Figure 2, imbalanced photocarriers transport along the electric field lines between anodes and cathodes [6], integrating into a rugby ball-like beam. Moreover, this is assuming only electrons are generated and participate in Drift–Diffusion motion.

Based on the direction of the photocarrier beam, E can be equivalent to three vector components $E_{//}$, $E_{⊥}$, and $E_{z}$. // and $E_{⊥}$ represent two directions respectively in parallel and perpendicular to the direction of photocarrier beam, while the z axis is for the one perpendicular to the plane formed by // and $E_{⊥}$. Spatial non-uniformity, time-dependence, and of field caused by built-in field are not taken into consideration. Thus, electron current through cut-planes near cathodes should be formulated as follow:

(3) $$I_{n_{cathode}}=\int \vec{J_{n}}d\sigma =qn\mu_{n}\int \left(\frac{\partial E\left(l_{x},l_{⊥}\right)}{\partial l_{z}}+\frac{\partial E\left(l_{x},l_{⊥}\right)}{\partial l_{⊥}}\right)dl_{z}dl_{⊥}\;+qD_{n}\int \left(\frac{\partial n\left(l_{x},l_{⊥},\;t\right)}{\partial l_{z}}+\frac{\partial E\left(l_{x},l_{⊥},\;t\right)}{\partial l_{⊥}}\right)dl_{z}dl_{⊥}$$

As the result of the rectangular geometry of the metal film for electrodes in PCSSs, E field keeps constant along z axis, thus equaling the partial derivative of E with respect to $l_{z}$ to zero, like $\frac{\partial E\left(l_{z},l_{⊥},t\right)}{\partial l_{z}}=0$. Then, Equation (4) can be simplified as:

(4) $$I_{r}=I_{cathode}=f_{1}\left(x,y,z\right)n\left(t\right)+f_{2}\left(x,y,z\right)n\left(t\right)$$

According to the test circuit, the electron current near the cathode equals the load current $I_{r}$. This means the load current should be linear in relation with the time-dependent function of free electrons. $f_{1}\left(x,y,z\right)$ and $f_{2}\left(x,y,z\right)$ are both independent from t, which stands for the parts not varying along with time in the Drift–Diffusion equation. So far, we have separated time-dependence and space-dependence. Moreover, these two functions are actually determined by the electric-field distribution on which the geometric structure of electrodes has a strong influence.

When SI 4H-SiC PCSSs with opposed electrodes shown in Figure 2a are illuminated by 532 nm laser [14,25,26], it is a linear extrinsic mode in which electron generation rate and photogeneration rate can be expressed as [27]:

(5) $$n\left(t\right)=\frac{\left(1-r\right)\left(1-e^{-\bar{\alpha }\mu }\right)}{\mu dLh\omega }\int_{-\infty }^{t}P\left(s\right)e^{\frac{\left(s-t\right)}{\tau }}ds$$

EOA occurs in several levels formed by impurities and defects, when the energy difference between them is smaller than the optical energy. So, as for a deep acceptor level whose electron exciting energy to conduction band is ΔE, the optical absorption is given by [3]:

(6) $$\alpha \left(h\omega \right)=N\sigma \left(h\omega \right)=N\frac{{\left(h\omega -\Delta E\right)}^{\frac{3}{2}}}{{\left(h\omega \right)}^{3}}$$

$\bar{\alpha }$ is a synthetic variable containing the doping levels and state density in EOA mode. To investigate the relation of temporal optical power, fit the experimental data collected by Amplified Photodetector with the gaussian function. From the fitting report, R-Square, called goodness of fit, 99.4% declares a fairly high similarity between original optical signal and fitted curve. This indicates the function of temporal optical power P(t) can reflect the time-dependent characteristics of triggering source on PCSSs. Equation (7) shows the time-dependent optical power of the light source.

(7) $$P\left(t\right)=\frac{A}{\bar{P}\times T}\times \left(-0.047+\frac{17.13}{5.15\times \sqrt{\frac{\pi }{2}}}\cdot e^{-\frac{{\left(t-8.538\right)}^{2}}{3.642^{2}}}\right)\left(W\right)$$

**Figure 3:**
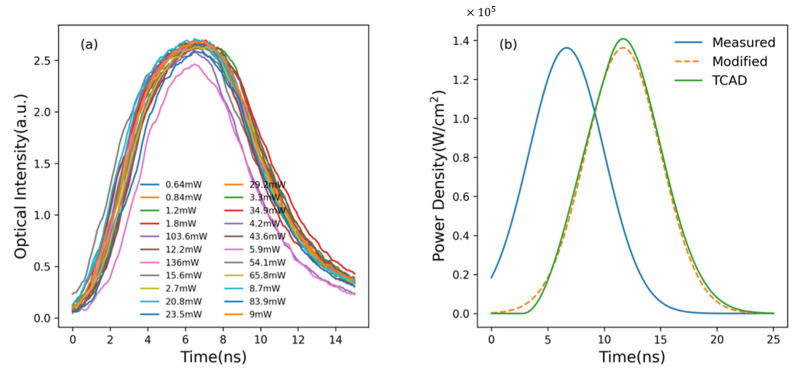
(**a**) Measured light signals; (**b**) the blue solid line is the averagely fitted curve by Equation (7); the orange dashed line, called modified light signal, comes from the blue line shifted by 5 ns along x axis, which is the rise delay of independent voltage source in Figure 5a; the green solid line represents an illumination signal in TCAD with adapting parameters of a piecewise gaussian function, as Equation (8), to the modified light signal.

Here, the first fraction item signifies the ratio from theoretical power to actual power of the optical source. $\bar{P}$ is the average power read by Power Meter. T is the triggering period which is the reciprocal of frequency. Figure 3 shows the measured light signals with various average powers and their fitted $P\left(t\right)-t$ curve.

(8) $$P\left(t\right)\left(TCAD\right)=\left\{\begin{array}{ll} i1, & if\;0<t<td1\\ \left(i2-i1\right)\left(1-exp\left(-{\left(\frac{t-td1}{tau1}\right)}^{2}\right)\right), & if\;td1<t<td2\\ \left(i2-i1\right)\left(1-exp\left(-{\left(\frac{t-td1}{tau1}\right)}^{2}\right)\right)-\left(i2-i1\right)\left(1-exp\left(-{\left(\frac{t-td2}{tau2}\right)}^{2}\right)\right), & if\;t>td2 \end{array}\right.$$

Since we created Equation (5) for dynamic photocarriers and Equation (7) for pulsed optical power, combine them into Equation (4) and then get the transient photocurrent of PCSS. With the assumption that the semi-insulator substrate has reached the state of thermal equilibrium before illumination beginning from t=0, the integral for t<0 in Equation (5) can be dismissed. Hence, the form of mapping relation between t and $I_{r}\left(t\right)$ has been discovered. Due to this mathematical structure, we tried two methods to simplify this mapping function. One is the gaussian method, because of the primary gaussian function fitted for optical signal, which has a critical influence on photoconduction behavior of PCSS [12]. The other is the polynomial method. The range of integration (0, t) in scale of ns is so small that Maclaurin expansion can be applied to simplify the integral term with $\int_{0}^{t}e^{x^{2}}dx$. Then, $I_{r}\left(t\right)$ can be approximated as a gaussian function as Equation (9) and as a quintic polynomial with t as Equation (10):

(9) $$I_{r}\left(t\right)\approx amp\times exp\left(\frac{{\left(t-\mu \right)}^{2}}{2\sigma^{2}}\right)$$

(10) $$I_{r}\left(t\right)\approx A5\times t^{5}+A4\times t^{4}+A3\times t^{3}+A2\times t^{2}+A1\times t^{1}+A0\times t^{0}$$

**Figure 4:**
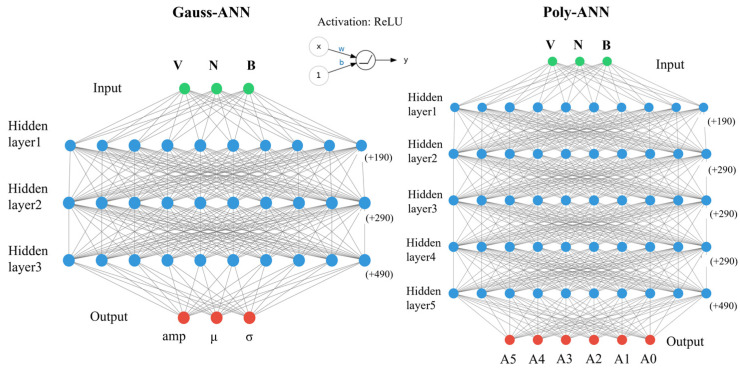
Architectures of Gauss-ANN and Poly-ANN.

In Equation (9), amp, μ and σ are, respectively, the amplitude, mean and standard deviation of a common gaussian function. In Equation (10), A5, A4, A3, A2, A1, A0 in turn mean the power of exponential items from degree 5 to 0 of variable t. Then, two types of models can be created based on these two fitting functions, named Gauss-ANN and Poly-ANN, depicted in Figure 4. The three-dimensional input array consists of doping concentration, while output is determined by the specific equation. Hidden layers of two architectures have been shown, but the neuron numbers are too big to be fully displayed. Both of the two models are made up of input layers with 200 neurons and output layers with 500 neurons. Gauss-ANN has three hidden layers while Poly-ANN has five, and the neuron number of each is 300, which will be further discussed later. The activation function is Rectified Linear Unit (ReLU), expressed as $max(0,w^{T}x+b)$. For linear functions, ReLU is more expressive, especially in deep networks; For nonlinear functions, ReLU does not have a vanishing gradient problem because the gradient of the non-negative interval is constant, so the convergence rate of the model is maintained in a stable state.

## 3. Simulation and Measurement

**Figure 5:**
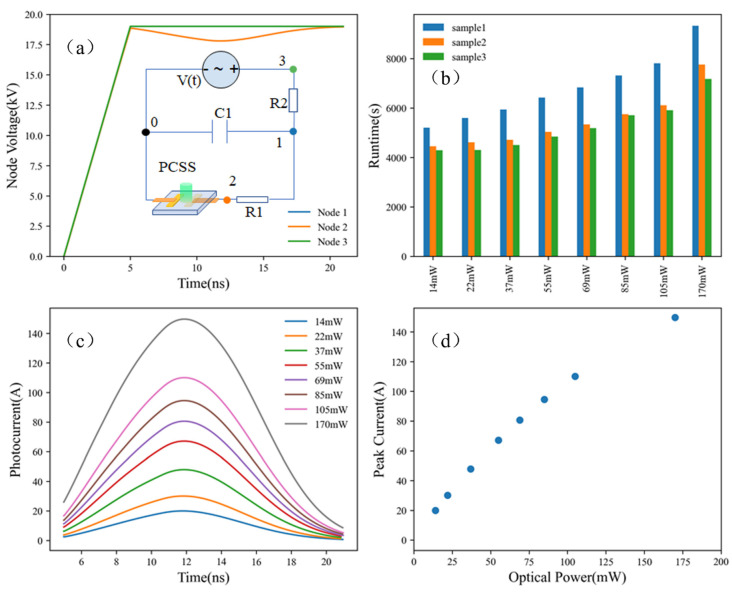
Mixed-Mode simulated (**a**) node voltage in circuit configuration, (**b**) runtime cost of three types of devices, (**c**) transient photocurrent, and (**d**) peak currents of ET-PCSS with opposed electrodes under various triggering optical powers.

Mixed-Mode is a circuit simulator that includes elements simulated by device simulation and a compact circuit model. It combines different levels of abstraction to simulate relatively small circuits where compact models for single devices are unavailable or insufficiently accurate. Physically based device simulation solves systems of equations that describe the physics of device operation. Figure 5 shows the Mixed-Mode simulated transient photoconductive properties of ET-PCSS with opposed electrodes under various triggering optical powers. Figure 5a shows V-t curves at each node in circuit configuration, whose topological structure is described in inset. In the same way, measurement of the transient properties of ET-PCSS was carried out by a high-voltage pulsed circuit with load. The input loop was made up of an independent voltage source V(t) of 19 kV, a current-limiting resistance R2 of 1 MΩ and a charge–discharge capacitance of 14.4 nF, while the output loop was made up of a PCSS and a load resistance R1 of 60.3 Ω. No more detailed description for the fabrication of the PCSS device is given here because of its unimportance in this article. In other words, any style of device is tolerable in theory. Two electrodes consisting of Ni (200 nm)/Ti (120nm)/Au (300 nm) were arranged diagonally across the switch. The voltage rises up to 19 kV over 5 ns and holds for 15 ns, which is set as the conducting time of the PCSS because it is triggered by a gaussian illumination beginning at 5 ns and ending at 20 ns. Figure 5c is the transient photocurrent during the conduction of the ET-PCSS device. Figure 5d extracts the peak current values form every I-t curve in (c). There seems to be a nonlinear relationship between them due to the saturation of optical absorption, discussed later.

This approach provides predictive capabilities and information about the conditions inside a device, but consumes significant amounts of CPU time. Given in Figure 5b, simulating the running of three PCSSs with different concentrations of vanadium, nitrogen, and boron dopants, refer to Table 1, costs several thousands of seconds. Additionally, the runtime increases when vanadium concentration increases. For example, the concentration of vanadium dopant in sample 1 reaches $2.174\times 10^{17}\;cm^{-3}$, more than that in sample 2 and 3, and as such, there is an extra 1000–2000 s of runtime. In addition, the optical power of triggering illumination also has an unfavorable effect on the runtime. In view of these facts, seeking another assisting method should save a lot of time cost for extracting properties of ET-PCSSs.

## 4. Sampling and Preprocessing

Obtaining data is an important step in training an ANN model. To this end, we use Latin Hypercube Sampling (LHS), which is a kind of layered Monte Carlo method suitable for multi-dimensional spaces when few samples are applied. The concentration of vanadium, nitrogen, and boron dopants are regarded as feature variables, forming a three-dimensional input space in which approximately 300 samples are enough. The sampling process is shown in Table 2. Firstly, we define the range for three impurities according to semi-insulated SiC material used as the substrate of the ET-PCSS. These ranges are merely on for partial wafers grown by specific methods rather than for all kinds of SiC crystals. Next, these sections should be divided into minizones based on the sampling numbers. Then, take out a random value from every minizone. Finally, assemble these values by three in a group without using any one value twice.

**Figure 6:**
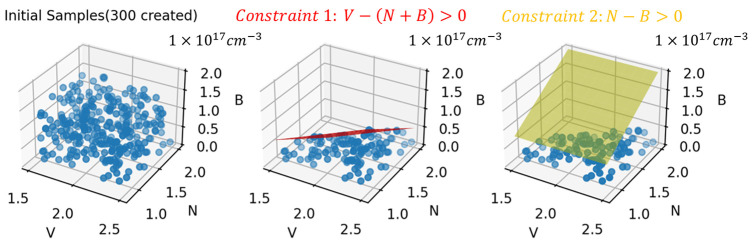
Schematic of constrained LHS. Red and yellow planes in 3D space represent constraints 1 and 2.

Also worth mentioning is the particularity in LHS processing. That is, the simulation solver could be non-convergent, or the doping concentration of samples may violate the fact in some cases. As a summary, it can be concluded as two constraints among three variables. One is when vanadium concentration (represented by V) is smaller than the sum of the concentration of nitrogen (represented by N) and boron (represented by B), 4H-SiC substrate of PCSSs will lose the characteristic of semi-insulating, leading to a large deviation from measurement. Another, if N is less than B, the simulation solver will not reach convergence, possibly as a result of the net doping of electrons being smaller than zero. Herein, a constrained LHS is implemented with two constraints for higher feasibility of sampling. Details can be seen in Figure 6. The whole flow is divided into three steps. First is a regular LHS based on the range of variables, after which 300 samples are created. The second step is partitioning the 3D space with a red plane formulated by the constraint 1 $\left(V-\left(N+B\right)>0\right)$. The third is similar to step 2, with a yellow plane formulated by the constraint 2 $\left(N-B>0\right)$. Finally, 108 filtered samples were ready for simulation and model training.

## 5. Results and Discussion

**Figure 7:**
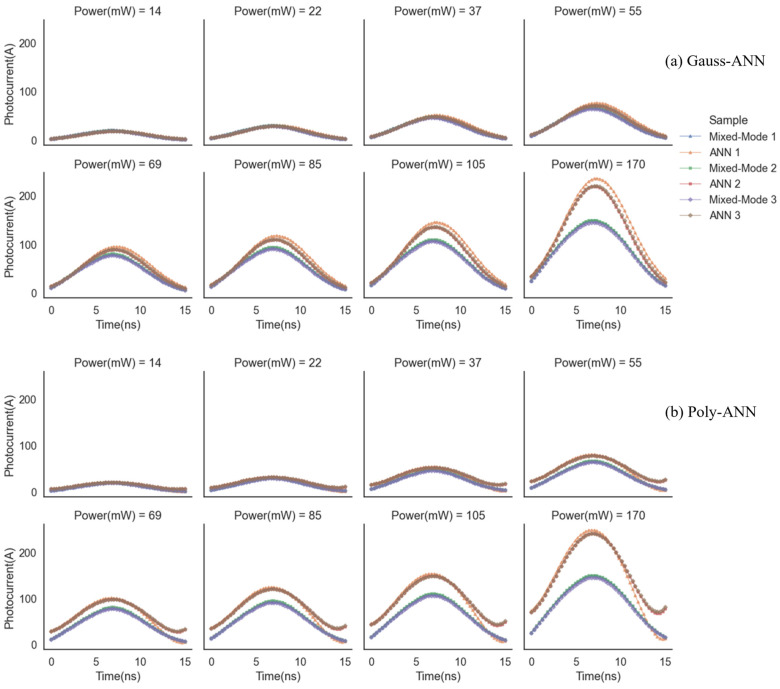
Single-pulsed transient characteristics of ET-PCSSs in Table 1, forecasted by (**a**,**b**). In the legend, two of the same symbols with dissimilar colors belong to the same species of PCSS, respectively by ANN forecasting and Mixed-Mode simulating.

As shown in LHS processing, both constraints take the doping concentration as a three-dimensional input of ANN, while the predicting ends are parameters of in fitting equations for two models. This means the whole monopulsed transient characteristics of an ET-PCSS can be described as long as all the parameters are forecasted by trained ANN predictors. In Figure 7, photocurrents–time response curves under various triggering powers of the three ET-PCSSs in Table 1 are presented. Here, we compare the transient currents of three devices calculated from Mixed-Mode and ANN models (Gauss-ANN shown in Figure 7a and Poly-ANN shown in Figure 7b). From the perspective of forecast accuracy, mean absolute error (MAE) of sample 1, for example, under various triggering powers is successively 2.0 A, 3.1 A, 5.4 A, 8.8 A, 12.1 A, 16.4 A, 22.4 A, and 47.2 A in the Gauss-ANN predictor, while being 1.4 A, 2.6 A, 5.7 A, 10.3 A, 14.5 A, 19.8 A, 27.3 A, and 56.1 A in the Poly-ANN predictor. At low powers such as 14 mW, 22 mW, 37 mW, and 55 mW, the predicting outcomes are more believable for MAE of photocurrent less than 10 A. The higher the triggering power is, the more deviation in transient photocurrent there is. Wei et al. [16] investigated the switching transient of GaAs PCSS undergoing the transition from a linear mode to a nonlinear mode, based on a “two-channel” model. Simulations and experiments reached an agreement on the formation and evolution of multiple avalanche domains by comparing the output waveforms under different triggering energies, which verifies the effectiveness of the proposed physical model. This “two-channel” model is a kind of physics-based model that belongs to GaAs PCSSs with linear and nonlinear modes at the same time. In a similar way, our work built a transient model for ET-PCSS in its only linear mode, preparing for a physics-based ANN architecture. Both simulations and experiments revealed a great degree of fitting for transient curves of ET-PCSSs. What is worth mentioning is that in quintic polynomial fitting based Poly-ANN predictors, loss of robustness is due to the complexity of polynomial coefficients, compared with gauss fitting. Therefore, a curl of tail the in photocurrent-time curve can be found in the Poly-ANN model, especially under higher powers, but there is little difference in peak current between the two models. In addition, total time cost in the Gauss-ANN and Poly-ANN model is 6.0 s and 4.5 s, including training and predicting, which is much lower than the several thousands of seconds for pure TCAD simulation.

**Figure 8:**
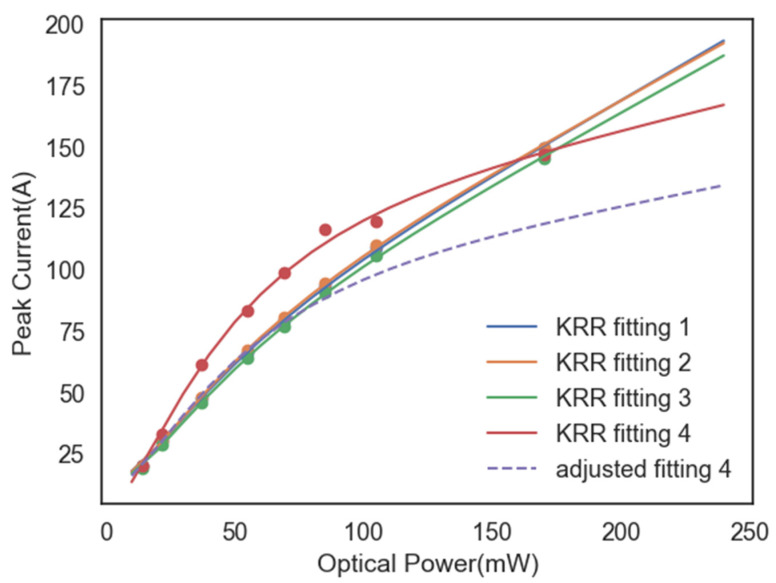
KRR fitting relationship between peak currents and optical powers of three samples from Gauss-ANN and one from experimental test. The dashed line stands for the relationship curve adjusted based on the lower optical powers of the experimental relationship, which can be applied for estimating higher optical powers in ANN models.

To verify the results of ANN models, Kernel Ridge Regression (KRR) is introduced to fit peak photocurrents at different optical powers, shown in Figure 8. Take the Gauss-ANN predictor as an example; KRR fitting relations of the three samples in Table 1 are similar exactly as their transient properties are also close. Compared to the experimental test, named KRR fitting 4, there is a minimum in peak deviation at 14 mW and 170 mW. This seems to be unusual with the monopulsed transient characteristics described previously. In fact, the cause is that light absorbing saturation is neglected both in TCAD simulation and ANN predictors. For the interface between the metal and the semiconductor, specific contact resistance is the key factor leading to the photoelectric saturation effect. Generally, the On-resistance of PCSS device can constitute two contact resistances between metal and semiconductor and an illuminating resistance by semiconductor. As a result, a more reliable relationship should be adjusted based on peak values at lower optical powers of the experimental relationship, i.e., adjusted fitting 4, so the contact resistance will play a leading role in the device resistance in which case photocurrent will not change with the increase of optical power. That is what should be optimized in physics-based ANN models as well as TCAD simulation in the future.

**Figure 9:**
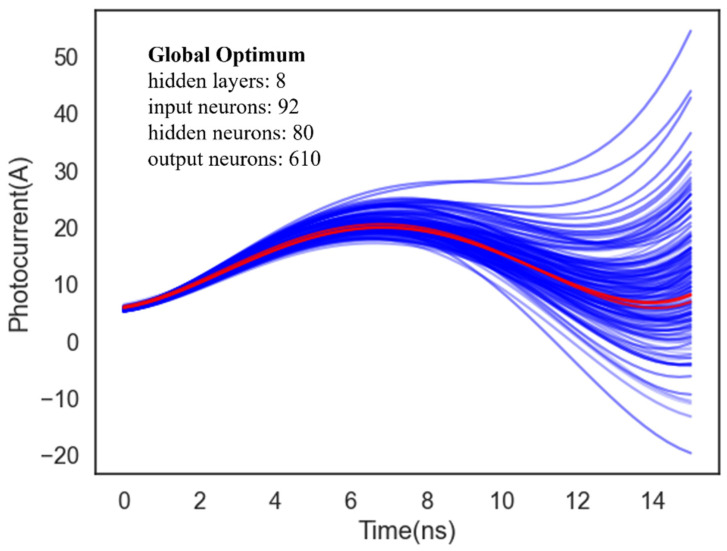
Multiple iterations during Bayesian Optimizing for Poly-ANN predictor. 100 blue lines are the acquisition points according to Bayesian algorithm, then the red line represents a global optimum about curl deviation.

Initial architectures of Gauss-ANN and Poly-ANN models are configured with three input variables, input layers with 200 neurons, three hidden layers with 300 neurons and an output layer with 500 neurons. By comparation, the randomness in model converging has a greater influence on the latter, which mainly falls in the tail of the curve of photocurrent-time curves as mentioned previously. In order to weaken this phenomenon, Bayesian optimization (BO) is implemented for the objective of minimizing the deviation of the tail of curve. BO algorithm has certain advantages, especially for high-overhead black-box function optimization (such as machine learning model tuning). It enables efficient and accurate global optimization by selecting the next sampling point based on the confidence of existing data and model construction. The process of optimizing is given in Table 3. As can be seen in Figure 9, 100 iterations of transient curves from the Poly-ANN predictor are depicted with blue lines. Then, the red line among them is just the optimum for its smallest deviation from the actual transient photocurrent. This optimized model consists of eight hidden layers and 92 neurons of input layers, 80 neurons of hidden layers, and 610 neurons of output layers. Compared with the initial architecture, the depth of the ANN has increased, adding some nonlinearity between inputs and outputs. Nevertheless, more neurons of the output layer may enlarge the complexity and reduce the generalization ability, or even raise the time cost of ANN predictor.

Furthermore, the ANN architectures proposed in this article may have some limitations due to the fact that they came from the specific functional forms, especially for their application in more general photoelectric devices. There are always higher dimensions of parameters in these predictors, not merely concentrations of dopants, but also light source, geometry, evenly integrated modules and so on. It is apparent that a simple learning model is no longer enough. The priority is to improve the ability of generalization of models. For example, Wei et al. [28] regulated the switching speed in PCSS arrays by introducing additional inductances, which is interpreted with the theory of multiple avalanche domains. Experiments and simulations both demonstrated inductance modulation on a two-channel bulk GaAs PCSS array. The transient Current–Time curves are gaussian-like functions with different peak values and shapes determined by adjustable inductances. To some extent, this agrees with our physics-based transient model for ET-PCSS. However, inductance of external circuits is a key factor that influences the transient characteristics of a PCSS or PCSS array, which should be taken into account in high-frequency applications. Chen et at. [29] reported the effect of optical pulse width on the transient characteristics of GaAs PCSS. The rapid increase in electron concentration and impact ionization rate under the narrower pulse makes it easier to boost the GaAs PCSS into high-gain mode, which means a smaller trigger energy at same incident optical power. There is a guiding significance on the future development of the proposed technology of this work. That is, pulse width is critical to the transient property of a PCSS in the aspects of electron concentration and impact ionization rate. It is necessary to take pulse width into consideration for building models. As long as we increase the dimensions of the input array, the model becomes more powerful for PCSSs in different applications. It would be an exciting thing if we had a large model that covered all types of PCSSs no matter what they are made of or what the application scenario is. It may take only several minutes to obtain the transient performance of a PCSS with a known doping concentration under arbitrary operating conditions for wavelength, voltage, power and pulse width. However, the challenges are also unavoidable because of the various shapes of optical pulse. We should integrate sufficient types of functions into models to fit all kinds of transient curves. In addition, the mechanism of operating a PCSS involves light, electricity, heat and other physical fields. It ought to be a complicated problem including many coupling effects such as photoelectric saturation, irradiation heat and electromagnetic heat. So, a more powerful model based on complex physics becomes necessary in consideration of the fields above. It not only has the ability of predicting, but also classifying, searching and so on.

## 6. Conclusions

In this paper, we proposed a brand-new physics-based ANN assisting method for extracting the transient properties of ET-PCSSs. On the basis of theories for semiconductor devices, two branch models were established by two types of characteristic fittings, called Gauss-ANN and Poly-ANN. The MAE of forecasting could be less than 10 A when triggering optical power was inferior to 55 mW, which is attainable in the area of ultrahigh voltage devices and power electronics. Compared to Mixed-Mode simulation, the ANN approach saved thousands of seconds of time cost for each CPU run on the premise of calculating accuracy. Deviation of peak currents under higher optical powers was principally contributed to the effect of light absorbing saturation in the 4H-SiC substrate. We also adopted the BO algorithm to optimize the architecture of Poly-ANN for its lack of robustness.

## Figures and Tables

**Table 1:** Concentration of Dopants in different ET-PCSSs.

	Sample 1	Sample 2	Sample 3
Vanadium	$2.174\times 10^{17}\;{cm}^{-3}$	$1.617\times 10^{17}\;{cm}^{-3}$	$1.378\times 10^{17}\;{cm}^{-3}$
Nitrogen	$8.62\times 10^{16}\;{cm}^{-3}$	$8\times 10^{16}\;{cm}^{-3}$	$8\times 10^{16}\;{cm}^{-3}$
Boron	$1\times 10^{16}\;{cm}^{-3}$	$6.18\times 10^{16}\;{cm}^{-3}$	$5.81\times 10^{16}\;{cm}^{-3}$

**Table 2:** Algorithm: Latin Hypercube Sampling.

Step 1:	Determine the number of datasets (N=300) and ranges of doping concentration: $vanadium\in \left[1.0,2.5\right]\times 10^{17}\;{cm}^{-3}$, $nitrogen\in \left[0.8,2.0\right]\times 10^{17}\;{cm}^{-3}$, $boron\in \left[0.1,1.5\right]\times 10^{17}\;{cm}^{-3}$;
Step 2:	Divide into minizones as $\frac{1}{N}\left[a_{i},b_{i}\right],\;i=1,2,3$;
Step 3:	Get N random values from every minizone as $\left[a_{i}+\frac{k-1}{N}\left(b_{i}-a_{i}\right),a_{i}+\frac{k}{N}\left(b_{i}-a_{i}\right)\right],\;k\in [1,N),\;i=1,2,3$;
Step 4:	Randomly combine N random values of every variable with others under the principle that each value ought to be taken and only taken once.

**Table 3:** Algorithm: Bayesian optimization.

Step 1:	$H=\left(x_{1:k},f\left(x_{1:k}\right)\right)$,
Step 2:	For k ← 1 to K
Step 3:	$x^{*}\leftarrow \;{argmin}_{x}S(x,M_{k-1}),$
Step 4:	Evaluate $f\left(x^{*}\right)$,
Step 5:	$H\leftarrow H\cup \left(x^{*},f\left(x^{*}\right)\right)$,
Step 6:	Fit a new model $M_{k}$ to H.
Step 7:	return H

## Data Availability

All data, models, and code generated or used during the study appear in the submitted article.
